# APOE2 AND AGING-RELATED OUTCOMES IN 261,000 UK BIOBANK OLDER ADULTS

**DOI:** 10.1093/geroni/igz038.811

**Published:** 2019-11-08

**Authors:** Chia-Ling Kuo, Luke C Pilling, George A Kuchel, David Melzer

**Affiliations:** 1 University of Connecticut, Farmington, Connecticut, United States; 2 University of Exeter, Exeter, England, United Kingdom; 3 UConn Health, Farmington, Connecticut, United States

## Abstract

ApoeE2 (e2) has both protective and adverse effects on health outcomes, which needs to be considered when targeting e2. We aimed to understand the role of e2 in aging via associations between e2 and a selection of aging traits using UK older adults of European descent. e2e3 (n=32,262) and e2e2 (n=1,629) were compared to e3e3 (n=153,567), adjusted for sex, age, genotyping arrays, assessment centers, and the first five genetic principal components. We found that e2 was associated with both parents top 10% survival (OR=1.14, 95% CI: 1.06-1.24), reduced risks of hypertension (OR=0.93, 95% CI: 0.91-0.96) and coronary heart disease (OR=0.86, 95% CI: 0.82-0.89), but red cell width distribution above the reference range (OR=1.17, 95% CI: 1.09-1.26). Additionally, e2 was minimally associated with frailty, cognitive function, and physical measures, suggesting that its association with parental extreme longevity may be controlled by mechanisms underlying diseases.

